# Fugong virus, a novel hantavirus harbored by the small oriental vole (*Eothenomys eleusis*) in China

**DOI:** 10.1186/s12985-016-0483-9

**Published:** 2016-02-16

**Authors:** Xing-Yi Ge, Wei-Hong Yang, Hong Pan, Ji-Hua Zhou, Xi Han, Guang-Jian Zhu, James S. Desmond, Peter Daszak, Zheng-Li Shi, Yun-Zhi Zhang

**Affiliations:** Key Laboratory of Special Pathogens, Wuhan Institute of Virology, Chinese Academy of Sciences, Wuhan, 430071 China; Yunnan Provincial Key Laboratory for Zoonosis Control and Prevention, Yunnan Institute of Endemic Diseases Control and Prevention, Dali, 671000 China; EcoHealth Alliance, New York, NY 10001 USA

**Keywords:** *Eothenomys eleusis*, Hantavirus, Hemorrhagic fever with renal syndrome, Genome, Vole

## Abstract

**Background:**

Rodents are natural reservoirs of hantaviruses, which cause two disease types: hemorrhagic fever with renal syndrome in Eurasia and hantavirus pulmonary syndrome in North America. Hantaviruses related human cases have been observed throughout Asia, Europe, Africa, and North America. To date, 23 distinct species of hantaviruses, hosted by reservoir, have been identified. However, the diversity and number of hantaviruses are likely underestimated in China, and hantavirus species that cause disease in many regions, including Yunnan province, are unknown.

**Results:**

In August 2012, we collected tissue samples from 189 captured animals, including 15 species belonging to 10 genera, 5 families, and 4 orders in Fugong county, Yunnan province, China. Seven species were positive for hantavirus: *Eothenomys eleusis* (42/94), *Apodemus peninsulae* (3/25), *Niviventer eha* (3/27), *Cryptotis montivaga* (2/8), *Anourosorex squamipes* (1/1), *Sorex araneus* (1/1), and *Mustela sibirica* (1/2). We characterized one full-length genomic sequence of the virus (named fugong virus, FUGV) from a small oriental vole (*Eothenomys eleusis*). The full-length sequences of the small, medium, and large segments of FUGV were 1813, 3630, and 6531 nt, respectively. FUGV was most closely related to hantavirus LX309, a previously reported species detected in the red-backed vole in Luxi county, Yunnan province, China. However, the amino acid sequences of nucleocapsid (N), glycoprotein (G), and large protein (L) were highly divergent from those of *Hantavirus* LX309, with amino acid differences of 11.2, 15.3, and 12.7 %, respectively. In phylogenetic trees, FUGV clustered in the lineage corresponding to hantaviruses carried by rodents in the subfamily Arvicolinae.

**Conclusions:**

High prevalence of hantavirus infection in small mammals was found in Fugong county, Yunnan province, China. A novel hantavirus species FUGV was identified from the small oriental vole. This virus is phylogenetic clustering with another hantavirus LX309, but shows highly genomic divergence.

## Background

Hantaviruses (genus *Hantavirus*, family *Bunyaviridae*) cause two disease types: hemorrhagic fever with renal syndrome (HFRS) in Eurasia and hantavirus pulmonary syndrome (HPS) in North America [1]. The hantavirus genome is composed of three segments, designated the large (L), medium (M), and small (S) segments, which encode an RNA-dependent RNA polymerase [2, 3], a glycoprotein precursor that is cotranslationally processed to produce two envelope glycoproteins (Gn and Gc), and a nucleocapsid (N) protein, respectively [3, 4]. HFRS is a typical rodent-borne disease and a severe public health issue in China [5]. In addition to rodents, hantaviruses have recently been detected in shrews (order Soricomorpha, family Soricidae and Talpidae) [6–11] and bats (order Chiroptera, family Rhinolophidae, Hipposideridae, Vespertilionidae, and Nycteridae) [12–16], but it is not clear whether these hantaviruses lead to human illness. It is believed that only hantaviruses in rodent hosts (order Rodentia, family or subfamily Muridae, Arvicolinae, and Sigmodontinae) cause HFRS and HPS in Asia, Europe, Africa, and North America [17]. To date, 23 distinct hantavirus species are recognized by the ICTV [18]. In China, two main hantaviruses, Hantaan virus (HTNV) carried by the striped field mouse (*Apodemus agrarius*) and Seoul virus (SEOV) carried by the Norway rat (*Rattus norvegicus*), cause HFRS [19]. In 2012, we reported a novel hantavirus harbored by the Yunnan red-backed vole (*Eothenomys miletus*) [20]. In this study, we identified another novel hantavirus in the small oriental vole (*Eothenomys eleusis*, subfamily *Arvicolinae*), designated Fugong virus (FUGV) because it was recovered in Fugong county, Yunnan Province, China. The small oriental vole is distributed widely in Hunan, Hubei, Sichuan, Yunnan, and Guizhou, China [21].

## Methods

### Animal sampling

In August 2012, 189 small mammals were captured in forests of the suburbs of Fugong county, Yunnan province. Animal species were identified based on morphology, and animal weight and sex were recorded. Species were further identified by DNA sequencing of the mitochondrial cytochrome b (*CytB*) gene following previously described methods [22]. Animal lung tissues were collected for further analysis.

### Direct immunofluorescence assay (IFA) detection of hantavirus

Hantavirus antigens present in the lung tissue samples were detected by direct immunofluorescence assay (IFA) as previously described [23]. Briefly, animal lung tissue samples were fixed and cut into 4-μm sections using a Cryocut microtome (KD-1508 Frigocut; Zhejiang, China) at −25 °C and fixed in cold acetone. Sections were stained with rabbit anti-SEOV/Z37 or HTNV/Z10 antibodies labeled with fluorescein isothiocyanate (FITC) (Zhejiang CDC) [24]. Scattered, granular fluorescence in the cytoplasm was considered positive staining.

### DNA and RNA extraction and reverse transcription PCR

Total DNA was extracted using the DNeasy Blood & Tissue Kit (Qiagen, Hilden, Germany) from tissue samples according to the manufacturer’s protocol. Viral RNA was extracted from lung tissues using the QIAamp Viral RNA Mini Kit (Qiagen) following the manufacturer’s instructions, and cDNA was synthesized with M-MLV reverse transcriptase (Promega, Madison, WI, USA) in the presence of the primer P14 [25].

Tissues from positive samples were screened by reverse transcription PCR (RT-PCR) using degenerate primers based on the conserved domain of the L fragment of *Hantavirus* genomes [26]. Standard precautions were taken to avoid PCR contamination, and no false-positives were observed in negative controls. PCR products were gel purified and sequenced with both forward and reverse primers using the 3100 Sequencer (ABI, Waltham, MA, USA).

### Genomic sequencing

One positive sample of *E. eleusis* was chosen for hantavirus genomic sequencing. The complete genomic sequence was amplified by PCR using primers designed from published hantavirus sequences or from sequences obtained in this study (available upon request). PCR products were also gel purified and sequenced with both forward and reverse primers using the 3100 Sequencer (ABI). The sequencing chromatograms were inspected carefully for overlapping multicolor peaks, which are an indicator of sequence heterogeneity in the amplicons. The PCR products of these samples were cloned into the pGEM-T Easy Vector (Promega) and at least 5 clones for each PCR fragment were sequenced to obtain a consensus sequence.

### Sequence analysis

Preliminary sequence management and analysis were carried out using Geneious (Version 5.5.9, Biomatters Limited, Aukland, New Zealand) and sequence alignment and editing were performed using ClustalW (Version 2.0) [27] and BioEdit (Version 7.1.9) [28]. The potential in vitro recombinant sequences (i.e., PCR artifacts) were screened and discarded using Recombination Detection Program v2.0 [29]. The consensus sequences were compared with known hantavirus sequences available in GenBank. Phylogenetic trees were constructed using the maximum likelihood (ML) algorithm with bootstrap values determined by 1000 replicates in Geneious.

## Results

### Detection of hantaviruses in several animals

**Fig. 1:**
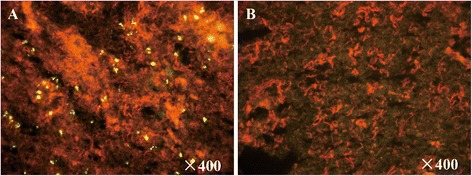
Detection of hantavirus in lung samples of *Eothenomys eleusis* by immunofluorescence. **a**: positive; **b**: negative. Yellow granular dots represent positive staining

The sampled animals consisted of 15 species belonging to 10 genera, 5 families, and 4 orders. A total of 49.74 % of captured animals (94/189) were *E. eleusis*, indicating that it may be the dominant species in the sampling area in Fugong (*CytB* GenBank No: KT899700). A summary of the species observed in the study is provided in Table 1. Based on IFA for all lung tissue samples (Fig. 1), hantaviruses were detected in 7 species: *Eothenomys eleusis* (42/94), *Apodemus peninsulae* (3/25), *Niviventer eha* (3/27), *Cryptotis montivaga* (2/8), *Anourosorex squamipes* (1/1), *Sorex Araneus* (1/1), and *Mustela sibirica* (1/2) (Table 1).

**Table 1:** Detection of hantavirus in small mammals in Fugong County, Yunnan Province, China in 2012

Animal species	Virus Counts (Positive/Tested)	
	IFA	Nested RT-PCR
Rodentia, Cricetidae, Eothenomys		
*Eothenomys eleusis*	42/94	21/94
Rodentia, Muridae, Apodemus		
*Apodemus peninsulae*	3/25	0/25
*Apodemus latronum*	0/9	0/9
*Apodemus chevrieri*	0/4	0/4
*Apodemus draco*	0/1	0/1
Rodentia, Muridae, Niviventer		
*Niviventer eha*	3/27	0/27
*Niviventer coxingi*	0/2	0/2
Rodentia, Muridae, Vernaya		
*Vernaya fulva*	0/1	0/1
Soricomorpha, Soricidae, Crocidura		
*Crocidura attenuata*	0/7	0/7
Soricomorpha, Soricidae, Cryptotis		
*Cryptotis montivaga*	2/8	0/8
Soricomorpha, Soricidae, Anourosorex		
*Anourosorex squamipes*	1/1	0/1
Soricomorpha, Soricidae, Sorex		
*Sorex Araneus*	1/1	0/1
*Sorex alpinus*	0/4	0/4
Lagomorpha, Ochotomidae, Ochotona		
*Ochotona thibetana*	0/3	0/3
Carnivora, Mustelidae, Mustela		
*Mustela sibirica*	1/2	0/2
Total	53/189	21/189

Hantavirus RNA was detected in 21 of 42 IFA-positive samples of *E. eleusis*. PCR amplification of the hantavirus sequence from samples obtained from 6 additional species failed. We sequenced all of the amplified fragments and found that the 420-nt (nucleotide) sequences were highly similar, with >99 % nt sequence identities. The hantavirus was named Fugong virus (FUGV).

### Genetic characterization and analysis

The full-length genomic sequence of FUGV from one positive sample obtained from *E. eleusis* (No.10) was determined (GenBank No: KT899701, KT899702, and KT899703). The full-length S-genomic segment was 1813 nt and encoded a predicted N protein of 435 aa; it contained noncoding regions (NCR) of 43 and 463 nt at the 5'- and 3'-regions, respectively (Table 2). The entire M-genomic segment was 3630 nt and encoded a predicted glycoprotein of 1190 aa; it contained NCRs of 58 and 152 nt at the 5'- and 3'-regions, respectively. For most hantaviruses, a conserved pentapeptide, WAASA, located at the end of Gn is presumed to be the proteolytic cleavage site to process the glycoprotein precursor into Gn and Gc [30]. Here, a variant WAVSA pentapeptide motif located at aa 648–652 was found. Moreover, using the NetNGlyc 1.0 server, six potential N-linked glycosylation sites in Gn and Gc at positions 136, 351, 403, 566, 579, and 931 were found.

**Table 2:** Nucleotide and amino acid sequence similarity (%) between FUGV virus and representative hantaviruses (23 ICTV-nominated species and two closely related viruses LX309 and YN06-862)

	nt and aa identities (%)					
	S segment		M segment		L segment	
Virus species (strain)	1813 nt	435 aa	3636 nt	1139 aa	6531 nt	2152 aa
*Hantavirus LX309*	63.1	88.8	75.7	84.7	76.3	87.3
*Hantavirus YN06-862*	91.1	99	-	-	-	-
*Andes virus Chile-9717869*	51.9	77.9	63.1	65.6	71.6	78
*Bayou virus F0260003*	53.4	78.3	62.2	64.5	70.8	77.7
*Black Creek Canal virus*	54	72.3	62.7	65.4	71.2	77.4
*Cano Delgadito virus VHV-574*	53.5	72.8	61.3	62.9	71.4	78.1
*Dobrava-Belgrade virus Afl9/1999*	45.3	60.9	56.9	54.6	66	68.8
*El Moro Canyon virus RM-97*	52.2	75.6	60.0	66.3	-	-
*Hantaan virus 76–118*	43.7	62.7	57.2	55.2	66.3	67.9
*Isla Vista virus MC-SB-1*	51.7	79	-	-	-	-
*Khabarovsk virus Fuyuan-Mm-312*	51.6	75.1	66.5	71	72.6	79.5
*Laguna Negra virus 510B*	52.6	75.8	62.5	64.9	-	-
*Muleshoe virus SH-Tx-339*	54.4	77.4	-	-	-	-
*New York virus NY-2*	53	74.9	62.9	65.6	-	-
*Prospect Hill virus PH-1*	50.9	77.4	67.5	71.5	70.6	79.5
*Puumala virus Sotkamo*	50.8	74	66.5	69.8	72.2	79.3
*Rio Mamore virus HTN-007*	52.6	77	63.2	65.6	70.9	78.3
*Rio Segundo virus RMx-Costa-1*	51.9	75.1	-	-	-	-
*Saaremaa virus Saaremaa-160v*	44.7	60.4	-	-	66.1	69.1
*Sangassou virus SA14*	44.8	61.6	56.6	53.8	66.6	67.8
*Seoul virus 80–39*	44.8	62.7	56.3	53.6	66.3	67.8
*Sin Nombre virus NM H10*	52.3	75.1	63.1	65.9	71.6	77.8
*Thailand virus Anjozorobe*	45.9	61.6	55.3	53.5	66	68.3
*Thottapalayam virus VRC 66412*	41.5	45.3	51.1	41.5	61.9	61.5
*Topografov virus Ls136V*	53.4	74.4	65	71.3	-	-
*Tula virus Moravia/5302v/95*	51.3	77	67.5	71.5	72.5	80

IFA, immunofluorescence analysis; RT-PCR, reverse transcription PCR

The full-length L-genomic segment was 6531 nt and encoded an RNA-dependent RNA polymerase of 2152 aa; it contained NCRs of 37 and 35 nt at the 5'- and 3'-ends, respectively. Previous studies have identified five conserved motifs (A, B, C, D, and E) in hantavirus RNA polymerases [31, 32]. These motifs were all detected in the FUGV L protein. In addition, other typical conserved motifs among hantavirus RNA polymerases, like premotif A and the acidic C-terminal domain [31], were found (data not shown).

A pairwise alignment and comparison with known hantavirus genomes (from previously detected species or strains) showed low nt sequence similarity in the S, M, and L segments, ranging from 41.5 to 63.1 %, 51.1 to 75.5 %, and 61.9 to 76.3 %, respectively (Table 2). Deduced aa similarities were higher (45.6–88.8 %, 41.5–84.7 %, and 61.5–87.3 % for the N, Gn/Gc, and L proteins, respectively). Additionally, all of the FUGV segments were most closely related to Hantavirus LX309 sequences, which is a putative novel hantavirus species found in the red-backed vole in Luxi County, Yunnan, China [20]. However, the aa sequences were highly divergent between the N, G, and L proteins of FUGV and Hantavirus LX309, with differences reaching 11.2, 15.3, and 12.7 %, respectively.

### Phylogenetic analyses

**Fig. 2:**
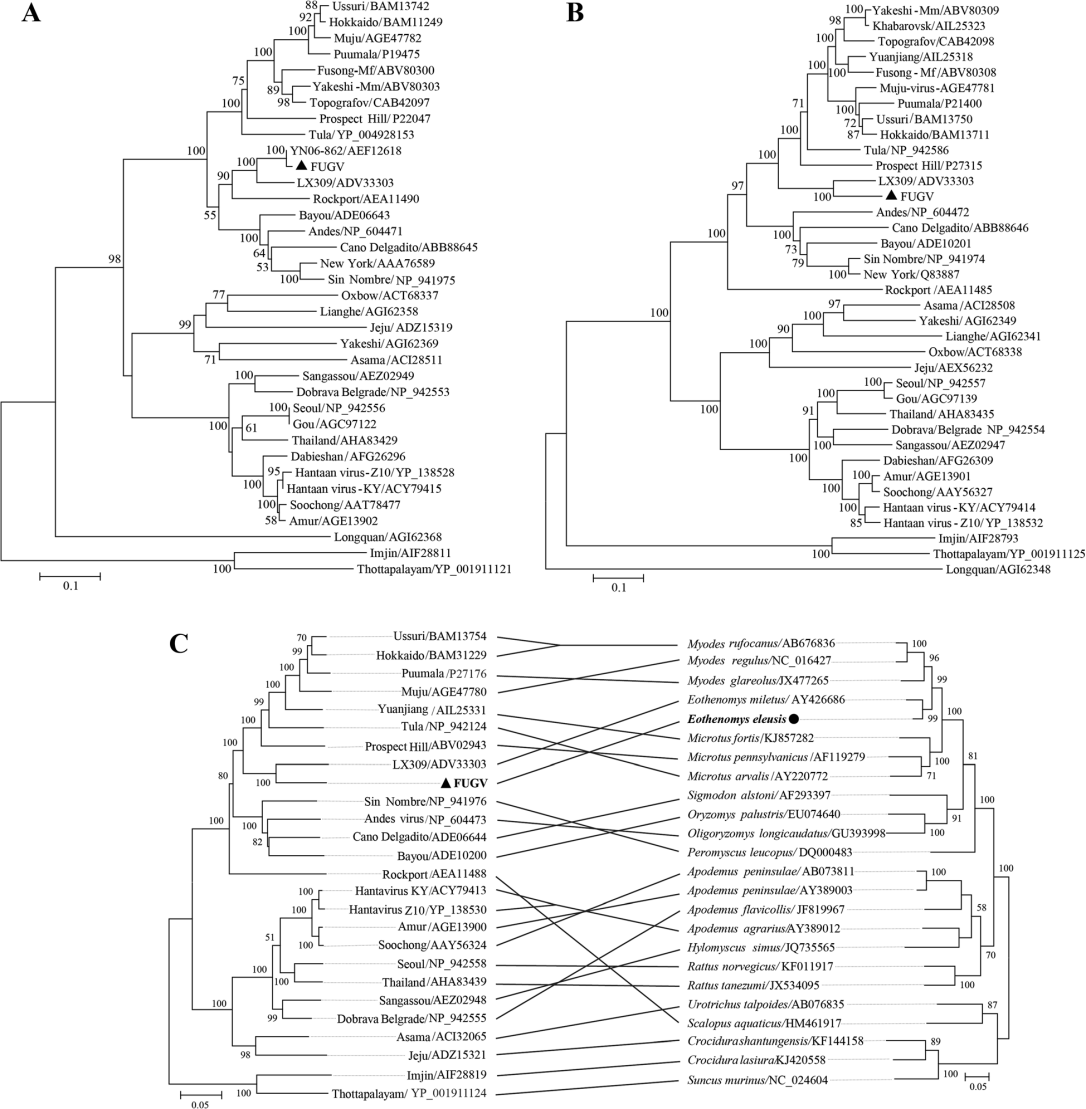
Phylogenetic analysis of hantaviruses and their hosts based on the L **a**, M **b**, and S segments and *CytB* **c**. Coding regions of the FUGV genome were aligned with sequences of representative hantavirus species (or strains) in GenBank, the alignments were used to generate ML trees, and the same method was used to build a *Cytb* tree. Bootstrap values greater than 50 % are shown at the branches. The GenBank accession numbers and the names of corresponding viruses are shown. FUGV and its *Eothenomys eleusis* host are labeled with triangles and circles, respectively

To determine the phylogenetic relationships among FUGV isolated in this study and previously described hantaviruses, the genomic segments of FUGV were aligned with those of representative hantavirus species (or strains) available in GenBank. Both nt and aa ML phylogenetic trees (based on coding regions and the predicted deduced protein sequences of the S, M, and L segments, respectively) were estimated. Either one or two genomic segments for several viruses were unavailable (e.g., the *El Moro Canyon* virus genome lacked the L region and the *Isla Vista* virus lacked M and L sequences); accordingly, the phylogenetic trees based on each genomic segment consisted of different numbers or combinations of viral taxa. Despite this, the nt and aa trees presented a consistent branching pattern and topology (Fig. 2). In the S, M, and L trees, FUGV clustered in the phylogenetic lineage that corresponded to viruses harbored by rodents in the subfamily Arvicolinae. In the M and L trees, FUGV and LX309 formed a distinct clade, both of which have been detected in the genus *Eothenomys* (Arvicolinae: Cricetidae) [20]. We noticed that another hantavirus in the database, YN06-862 (for which only the S segment was available in the database, and which lacked an associated publication, GenBank No. AEF12618), was most closely related to FUGV. YN06-862 was also detected in *E. eleusis* in Yunnan, China.

To infer the evolutionary relationships for FUGV and its host, an ML tree was built using mitochondrial *CytB* of the Rodentia hosts of representative hantaviruses, and Soricomorpha hosts were used as an outgroup (Fig. 2c). The tree of the hosts was in agreement with that of previous studies; the Murinae subfamily and Cricetidae family formed two monophyletic groups, with Soricomorpha as the outgroup. The Cricetidae were further subdivided into the subfamilies Arvicolinae, Neotominae, and Sigmodontinae [14]. To understand virus-host co-divergence, the L tree of viruses were compared with the *CytB* tree of hosts. We found that FUGV and LX309 diverged earlier than Prospect Hill virus, Tula virus, Puumala virus, and others, which was not congruent with their *Myodes* spp*.* host evolutionary statuses in the *CytB* tree. This phenomenon may be explained by differences in the evolutionary rates of hantaviruses in different hosts. In the tree, the same phenomenon can also can be observed for Sangassou virus and *Hylomyscus simus* (African wood mouse), and for Dobrava-Belgrade virus and *Apodemus flavicollis* (yellow-necked mouse) [33, 34].

## Discussion

Hantavirus antigens were detected by direct IFA in 7 out of 15 total species collected in Fugong county, Yunnan Province. We successfully amplified the nucleotide sequences of the virus obtained from *E. eleusis*. These results suggested a high cross-reactivity of SEOV and HTNV with other hantaviruses in serological level, but their genomic sequences were highly divergent. We thus suspected that there were other novel hantaviruses that cannot be detected with the degenerate primers used in this study. We also cannot exclude potential RT-PCR sensitivity issues.

The three segments of FUGV harbored by *E. eleusis* were most closely related to Hantavirus LX309 carried by *E. miletus*. However, the aa sequences of the N, G, and L proteins of FUGV were highly divergent with respect to Hantavirus LX309, with similarities reaching 11.2, 15.3, and 12.7 %, respectively. The new ICTV classification guidelines indicate that a novel *Hantavirus* species should show at least a 10 % difference in S segment similarity and a 12 % difference in M segment similarity based on complete amino acid sequences [35]. Accordingly, the hantavirus harbored by *E. eleusis* represents a novel species. Additionally, no reassortment or recombination events were detected among FUGV and other hantaviruses. We propose that this novel species be named Fugong virus (FUGV) based on the location where it was first recovered.

In phylogenetic analyses of the three segments of the FUGV genome, it grouped with hantaviruses harbored by rodents in the subfamily Arvicolinae, which can cause HFRS in Eurasia. For example, the *Puumala* virus (PUUV) harbored by Arvicolinae (*Myodes glareolus*) causes most HFRS cases, with 35,424 reported cases in 2006 [36, 37]. Recently, more than 100 HFRS cases have been reported in Yunnan province (unpublished data). However, the types of hantaviruses that cause HFRS in Yunnan province are still unknown. It is not clear whether FUGV can infect humans and lead to illness.

Phylogenetic analyses of hantaviruses and their rodent hosts suggested that the viruses have a long history of co-evolution with their predominant rodent carriers [38, 39]. FUGV was most closely related to hantavirus LX309, which is carried by different rodent species within the same genus. Additionally, we detected differences in the evolutionary rates of hantaviruses in different hosts. The *Eothenomys* genus consists of eight rodent species, but only two have been investigated. Considering the high prevalence and diversity of hantaviruses in the genus, additional investigations should be performed in the future to extend the number of species considered.
