# Erratum to: Fugong virus, a novel hantavirus harbored by the small oriental vole (Eothenomys eleusis) in China

**DOI:** 10.1186/s12985-016-0532-4

**Published:** 2016-05-05

**Authors:** Xing-Yi Ge, Wei-Hong Yang, Hong Pan, Ji-Hua Zhou, Xi Han, Guang-Jian Zhu, James S. Desmond, Peter Daszak, Zheng-Li Shi, Yun-Zhi Zhang

**Affiliations:** Key Laboratory of Special Pathogens, Wuhan Institute of Virology, Chinese Academy of Sciences, Wuhan, 430071 China; Yunnan Provincial Key Laboratory for Zoonosis Control and Prevention, Yunnan Institute of Endemic Diseases Control and Prevention, Dali, 671000 China; EcoHealth Alliance, New York, NY 10001 USA

## Erratum

Unfortunately, the original version of this article [1] contained an error. After publication it was noticed there was a missing acknowledgement. The end of the acknowledgment section should have stated the work was also jointly funded by the USAID Emerging Pandemic Threats (EPT) PREDICT program.

## References

[1] Ge X-Y, Yang W-H, Pan H, Zhou J-H, Han X, Zhu G-J, Desmond JS, Daszak P, Shi Z-L, Zhang Y-Z (2016). Fugong virus, a novel hantavirus harbored by the small oriental vole (Eothenomys eleusis) in China. Virol J..

